# Using Digital Tools to Engage Patients With Psychosis and Their Families in Research: Survey Recruitment and Completion in an Early Psychosis Intervention Program

**DOI:** 10.2196/24567

**Published:** 2021-05-31

**Authors:** Alexia Polillo, Aristotle N Voineskos, George Foussias, Sean A Kidd, Andreea Sav, Steve Hawley, Sophie Soklaridis, Vicky Stergiopoulos, Nicole Kozloff

**Affiliations:** 1 Centre for Addiction and Mental Health Toronto, ON Canada; 2 Department of Psychiatry University of Toronto Toronto, ON Canada

**Keywords:** digital, online, psychosis, schizophrenia, early psychosis intervention, surveys

## Abstract

**Background:**

Barriers to recruiting and retaining people with psychosis and their families in research are well-established, potentially biasing clinical research samples. Digital research tools, such as online platforms, mobile apps, and text messaging, have the potential to address barriers to research by facilitating remote participation. However, there has been limited research on leveraging these technologies to engage people with psychosis and their families in research.

**Objective:**

The objective of this study was to assess the uptake of digital tools to engage patients with provisional psychosis and their families in research and their preferences for different research administration methods.

**Methods:**

This study used Research Electronic Data Capture (REDCap)—a secure web-based platform with built-in tools for data collection and storage—to send web-based consent forms and surveys on service engagement via text message or email to patients and families referred to early psychosis intervention services; potential participants were also approached or reminded about the study in person. We calculated completion rates and timing using remote and in-person methods and compensation preferences.

**Results:**

A total of 447 patients with provisional psychosis and 187 of their family members agreed to receive the web-based consent form, and approximately half of the patients (216/447, 48.3%) and family members (109/187, 58.3%) consented to participate in the survey. Most patients (182/229, 79.5%) and family members (75/116, 64.7%) who completed the consent form did so remotely, with more family members (41/116, 35.3%) than patients (47/229, 20.5%) completing it in person. Of those who consented, 77.3% (167/216) of patients and 72.5% (79/109) of family members completed the survey, and most did the survey remotely. Almost all patients (418/462, 90.5%) and family members (174/190, 91.6%) requested to receive the consent form and survey by email, and only 4.1% (19/462) and 3.2% (6/190), respectively, preferred text message. Just over half of the patients (91/167, 54.5%) and family members (42/79, 53.2%) preferred to receive electronic gift cards from a coffee shop as study compensation. Most surveys were completed on weekdays between 12 PM and 6 PM.

**Conclusions:**

When offered the choice, most participants with psychosis and their families chose remote administration methods, suggesting that digital tools may enhance research recruitment and participation in this population, particularly in the context of the COVID-19 global pandemic.

## Introduction

While research is critically important to advancing the assessment and treatment of psychosis, conducting research with people with psychosis and their families can be challenging. Barriers to recruiting and retaining participants in psychosis research include study burden (eg, frequent and long study visits), illness severity, poverty, reluctance to accept diagnosis and treatment, low interest in participating among patients, and an unwillingness among care providers to refer patients to research [1-4]. Given the impairments associated with psychotic disorders and the challenges in recruiting and retaining participants [5-7], samples may be biased toward those who are higher functioning and more highly engaged in clinical services. Low recruitment rates are common and can impact study quality, resulting in small convenience samples or clinical trials that are not adequately powered [1,3,8-12]. When patients and families do participate in research, studies are vulnerable to high attrition rates and missed follow-up assessments, leading to missing data [1,12-14]. Together, these issues pose barriers to conducting robust clinical research with people with psychosis and their families.

Digital research tools, such as online platforms, mobile apps, and text messaging, have the potential to reduce barriers to conducting research with this population, improving recruitment and retention rates and leading to more robust, representative, and high-quality research. High rates of digital engagement have been found among young people, including those with psychosis [15-17], with email and text message cited as their preferred modes of communication [18]. Although evidence suggests that many people with psychosis own mobile phones and computers and have access to the internet [16,17,19-21], digital equity concerns remain for those living in poverty or experiencing homelessness [22]. Still, these technologies have been used to deliver mobile- and internet-based clinical interventions to those with psychosis, with substantial research examining their effects on symptoms, appointment attendance, and medication adherence [17,18,23]. However, few studies have examined how these technologies can be leveraged to engage people with psychosis in research. The body of research on use of electronic questionnaires does not specifically consider the preferences and digital literacy of people with serious mental illness [24]. Furthermore, given the lack of research in this area, institutional review boards (IRBs) tasked with assessing the risks associated with emerging digital tools have little guidance, which may lead to a slow approval process [25]. While their high rates of acceptability and utility as health interventions suggest that digital technologies may be effective research tools for people with psychosis and their families, there is little evidence to guide their use. This research gap has become even more apparent in the context of the COVID-19 global pandemic, where digital tools may offer a way to continue research that was postponed to accommodate physical distancing.

This exploratory paper examines the uptake of digital research tools among patients with psychosis and their families, as well as more traditional methods of recruitment and survey administration. We used digital research tools to obtain consent, administer a survey, and compensate patients and their families recruited at the time of their referral to early psychosis intervention (EPI) services. We also offered traditional alternatives, including in-person consent, a pen-and-paper survey option, and compensation with hard copy gift cards. The survey explored patient and family views on facilitators, barriers, and ideas to improve service engagement. These are critical aspects of EPI care given high rates of service disengagement [4] and gaps in the literature on patient- and family-reported perspectives [26]. Digital research tools were selected for their potential to remotely capture the perspectives of patients and families who were not well-engaged in clinical services. The objective of this study was to assess the uptake of digital tools to engage patients with provisional psychosis and their families in research, and their preferences for different research administration methods.

## Methods

### Overview

The Slaight Centre Early Intervention Service (SCEIS) is an EPI program at the Centre for Addiction and Mental Health in Toronto, Canada. Following the EPI model, it aims to provide comprehensive treatment delivered by a multidisciplinary team early in the course of illness [27]. It provides assessment for young people aged 16-29 years with provisional psychosis and offers treatment for up to 3 years for those with confirmed affective, nonaffective, and substance-induced psychosis. The program receives approximately 600 new referrals each year. Research is integrated into clinical care through a centralized and coordinated research recruitment process. Young people referred to the program between July 2018 and February 2020 and their family members were invited to complete a survey exploring patient- and family-reported facilitators and barriers to engagement in EPI services.

### Recruitment and Procedures

This study used Research Electronic Data Capture (REDCap)—a secure, web-based platform with built-in tools for data collection and storage—to send web-based consent forms and surveys to patients and families referred to EPI services [28-30]. REDCap self-sufficiently stores email addresses and phone numbers in a secure participant list to send forms directly by email or through SMS text message using a plug-in called Twilio. When the clinic administrator from SCEIS phoned to book the consultation appointment, verbal consent was sought from the patient to send them a link to a web-based consent form via text message or email. After obtaining verbal consent from the patient, when possible, the clinic administrator would ask to speak to a family member to send them the family consent form. Less typically, the family member was consented first if they booked the consultation appointment on behalf of the patient.

The web-based consent form, including a description of the survey's purpose and length, data storage, and investigator, as well as the purpose of the study, was sent after verbal consent was obtained. Two reminders were sent 2 days apart following the survey. In some cases, the appointment was arranged without a direct phone call (eg, by leaving voice-mail messages back and forth or emailing a discharge planner on the inpatient unit). Patients and family members who were missed for recruitment over the phone but presented in person were approached by the research team to explain the study and obtain web-based consent on a tablet or written consent on paper, depending on their preference. Participants who agreed to receive the consent form but had not yet consented were also approached in person. In addition to reminding patients and family members about the research project, in-person administration served to answer questions and provide technical support.

Web-based surveys were sent automatically through REDCap 30 days after participants consented to the study to capture early experiences in the EPI program. Surveys were approximately 12 pages in length, with 1-8 questions per page, and took less than 15 minutes to complete. If the survey was not completed remotely after 2 automated reminders, participants who were attending services were approached to complete the survey in person using a tablet or pen and paper, based on their preference. Participants were also contacted by phone and reminded to complete the survey. Those who completed the survey remotely were given the option to select a Can $10 (US $8.27) electronic gift (e-gift) card from their choice of a major chain of coffee shops, bookstores, or movie theatres upon survey completion; those who completed the survey in person were offered an e-gift or hard copy gift card. Before concluding the study, participants who had consented but not yet completed the survey were sent 2 automated messages notifying them that the study was ending and the link would be expiring. The recruitment process is outlined in Multimedia Appendix 1. In addition to answering questions about service engagement, participants were asked to self-report their sociodemographic characteristics according to locally developed standardized questions designed to capture health equity factors [31]. This study was approved by the Research Ethics Board at the Centre for Addiction and Mental Health and is reported in accordance with the Checklist for Reporting Results of Internet E-Surveys (CHERRIES; Multimedia Appendix 2 [32]).

### Data Analysis

We compiled descriptive statistics for consent and survey completion rates, the demographic characteristics of patients and family members who completed the survey, time and day of the week the survey was completed, and communication and gift card preferences. We also tracked the responses of participants who were contacted by phone to complete the survey. Data were analyzed with descriptive statistics including means, standard deviations, ranges, and percentages using Stata statistical software (StataCorp LLC [33]).

## Results

Consent and survey completion rates, as well as communication and gift card preferences, are outlined in Table 1. Of the 801 patients who were identified as being eligible for the study, 447 patients and 187 family members agreed to receive the consent form electronically from the clinic administrator or in person from the research team. Only 8 patients and 7 family members who were approached declined to receive the consent form; the remaining eligible patients were not reached by the clinic administrator directly (eg, they did not return calls to book their intake appointment or communicated by leaving voice mails back and forth), and if they came in person, they were missed by the research team. Approximately half of the patients (216/447, 48.3%) and family members (109/187, 58.3%) consented to participate in the survey. A small number of patients (13/447, 2.9%) and family members (7/187, 3.7%) responded to the consent form but did not wish to participate in the survey. Most patients (182/229, 79.5%) and, to a lesser extent, family members (75/116, 64.7%) who completed the consent form did so remotely as opposed to in person. Even when the consent form was administered in person, all patients and family members who participated in the study opted to complete it on a tablet in REDCap rather than using pen and paper.

**Table 1:** Consent and survey completion rates and digital preferences.

Completion rates and digital preferences		Patients		Family members	
		n (%)	N	n (%)	N
**Completion rates: consent form**					
	Yes	216 (48.3)	447	109 (58.3)	187
	No	13 (2.9)	447	7 (3.7)	187
	Did not respond	218 (48.8)	447	71 (38.0)	187
**Format in which consent form was completed**					
	Remote	182 (79.5)	229	75 (64.7)	116
	In person	47 (20.5)	229	41 (35.3)	116
**Completion rates: survey**					
	Yes	167 (77.3)	216	79 (72.5)	109
	Did not respond	49 (22.7)	216	30 (27.5)	109
**Survey nonresponders**					
	Partially completed survey	11 (22.4)	49	7 (23.3)	30
	Left survey blank or did not open it	38 (77.6)	49	22 (73.3)	30
	Had technical problems with REDCap^a^	0 (0)	49	1 (3.3)	30
**Format in which survey was completed**					
	Remote	139 (83.2)	167	68 (86.1)	79
	In person	28 (16.8)	167	11 (13.9)	79
**Communication preference**					
	Email	418 (90.5)	447	174 (91.6)	187
	Text message	19 (4.1)	447	6 (3.2)	187
	No preference	16 (3.5)	447	3 (1.6)	187
	Switched between preferences	9 (1.9)	447	7 (3.7)	187
**Gift card preference**					
	Coffee shop	91 (54.5)	167	42 (53.2)	79
	Bookstore	39 (23.4)	167	20 (25.3)	79
	Movie theatre	33 (19.8)	167	15 (19.0)	79
	Lost to follow-up or declined	4 (2.4)	167	2 (2.5)	79

^a^REDCap: Research Electronic Data Capture.

Of those who consented, 77.3% (167/216) of patients and 72.5% (79/109) of family members completed the survey. The demographic characteristics of patients and family members who participated in the study are highlighted in Table 2. Among the patients, the mean age was 22.8 years, 46.7% (78/167) were female, 63.5% (106/167) identified with racial groups other than White, and 63.5% (106/167) were born in Canada. Most patients (138/167, 82.6%) were single, 64.1% (107/167) were living with family, and 75.5% (126/167) had English as a first language. Almost half of the patients (80/167, 47.9%) attended or completed university, 33.5% (56/167) were in full-time work or school, and 25.7% (43/167) were receiving some form of income assistance. Over one-third of patients (54/167, 32.3%) reported weekly substance use in the past month.

Among family members, the mean age was 47.8 years, 59.5% (47/79) were mothers, 72.2% (57/79) lived with the patient, and 48.1% (38/79) identified with racial groups other than White. Half of the family members (40/79, 50.6%) were born in Canada, 67.1% (53/79) had English as a first language, and 62.0% (49/79) were married or in a relationship. Over one-third (32/79, 40.5%) of family members attended or completed university, 64.6% (51/79) were in full-time work or school, and 70.9% (56/79) were receiving earnings from regular work.

**Table 2:** Demographic characteristics of patients and family members who completed the survey.

Characteristics		Patients (N=167)	Family members (N=79)
Age (years), mean (SD)		22.8 (3.5)	47.8 (12.6)
Relationship to patient (mother), n (%)		N/A^a^	47 (59.5)
**Gender, n (%)**			
	Male	76 (45.5)	18 (22.8)
	Female	78 (46.7)	61 (77.2)
	Trans, nonbinary, 2-spirit, or unknown	13 (7.8)	0 (0)
**Racial group, n (%)**			
	East, South, or South East Asian	41 (24.6)	12 (15.2)
	Black African, Caribbean, or North American	23 (13.8)	13 (16.5)
	Latin American	11 (6.6)	6 (7.6)
	Middle Eastern	6 (3.6)	0 (0)
	White European or North American	61 (36.5)	41 (51.9)
	Other including First Nations, Inuit, Metis, or Indigenous/Aboriginal not included elsewhere; mixed heritage; Indian Caribbean; decline to answer; or do not know^b^	25 (15.0)	7 (8.9)
Born in Canada, n (%)		106 (63.5)	40 (50.6)
**Relationship status, n (%)**			
	Single, never married^c^	138 (82.6)	17 (21.5)
	Married, partner, or significant other	29 (17.4)	49 (62.0)
	Separated, widowed, or divorced	0 (0)	13 (16.5)
Living with the patient, n (%)		N/A	57 (72.2)
**Housing, n (%)**			
	Own an apartment/housing	26 (15.5)	N/A
	Live with parents or other family members	107 (64.1)	N/A
	Share a place with friends or peers	17 (10.2)	N/A
	Emergency shelter or couch surfing	4 (2.4)	N/A
	Supportive/transitional housing, treatment facility, or other	6 (3.6)	N/A
	Rooming or boarding house	7 (4.2)	N/A
English as a first language, n (%)		126 (75.5)	53 (67.1)
**Education, n (%)**			
	Attended or completed high school^d^	59 (34.3)	9 (11.4)
	Attended or completed college, or a trade or technical school	21 (12.6)	21 (26.6)
	Attended or completed university	80 (47.9)	32 (40.5)
	Attended or completed graduate school	7 (4.2)	17 (21.5)
Currently in full-time school or work, n (%)		56 (33.5)	51 (64.6)
Receiving earnings from regular work, n (%)		48 (28.7)	56 (70.9)
Receiving income assistance, n (%)		43 (25.7)	5 (6.3)

^a^N/A: not applicable.

^b^Respondents who answered “other”; First Nations, Inuit, Metis, or Indigenous/Aboriginal not included elsewhere; mixed heritage; Indian Caribbean; “decline to answer,” or “do not know” were grouped together to supress small cells.

^c^Respondents who were separated, widowed, or divorced were grouped in with “single, never married” to suppress small cells.

^d^Respondents who answered “other,” “don’t know,” or “decline to answer” were included in the group who attended or completed high school to suppress small cells.

**Figure 1:**
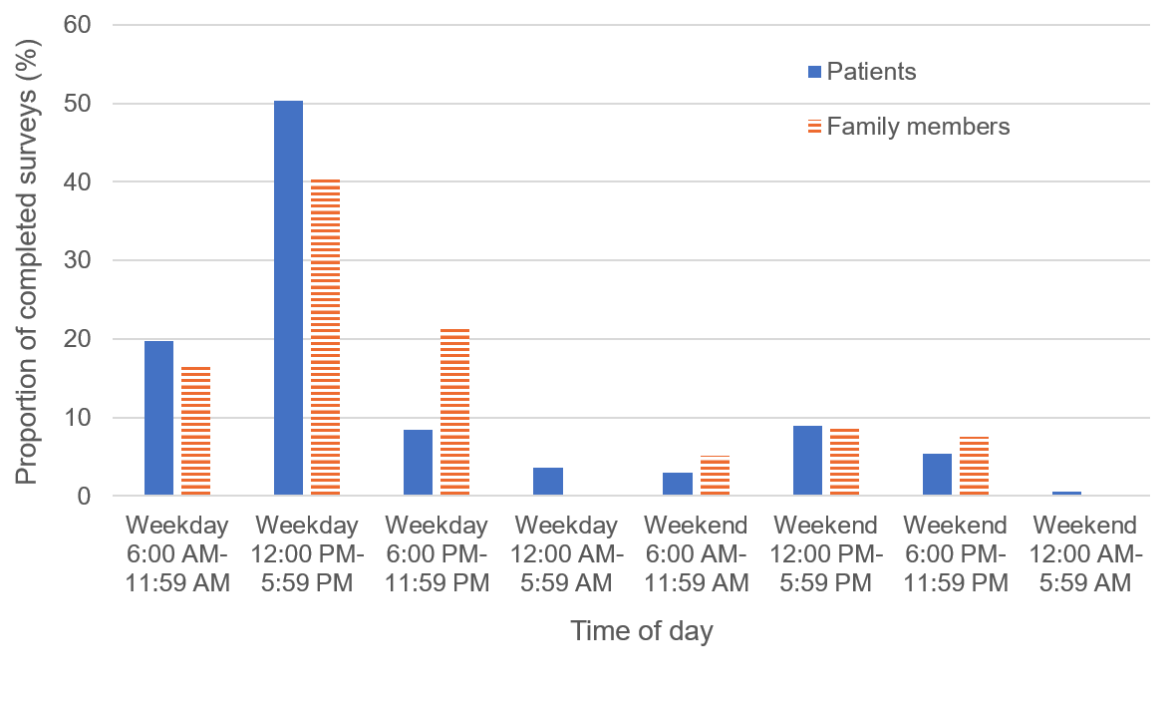
Timing of survey completion.

In total, 83.2% (139/167) of patients and 86.1% (68/79) of family members completed the survey remotely. Most surveys were completed on weekdays and more than half were completed between 12 PM and 6 PM, as shown in Figure 1. Almost all patients (418/462, 90.5%) and family members (174/190, 91.6%) requested to receive the consent form and survey by email, and only 4.1% (19/462) and 3.2% (6/190), respectively, preferred text message. Over half of the patients and family members preferred to receive e-gift cards from a coffee shop as study compensation (91/167, 54.5%, and 42/79, 53.2%, respectively). As with the consent form, all patients and family members who completed the survey in person opted to use a tablet rather than pen and paper.

Approximately one quarter of patients (49/216, 22.7%) and family members (30/109, 27.5%) did not complete the survey. Of these, 22.4% (11/49) of patients and 23.3% (7/30) of family members partially completed the survey, whereas the remainder did not open the survey or left it blank. One family member was not able to complete the survey because REDCap was having technical problems. Participants who had consented but not yet completed the survey were contacted by phone; those who were reached reported that they were either no longer interested in participating or asked to be sent the survey again. Participants who were unreachable by phone had voice mails that were not activated, phone numbers that were no longer in service, and incorrect contact information.

## Discussion

### Principal Findings

To our knowledge, this is one of the first studies to examine the use of digital tools to engage people with psychosis and their families in research. Patients and family members referred to EPI services were generally agreeable to receiving and completing the web-based consent form and survey and preferred these remote online administration methods to in-person and pen-and-paper–based methods. However, in-person engagement was still a useful strategy for reminding patients and family members about the research, explaining the research, and providing technical support. Patients and family members preferred to engage in research by email rather than text message and receive e-gift cards from a coffee shop as compensation for their participation. Our results suggest that digital tools can support effective recruitment, consenting, and survey administration among people with psychosis and their family members. They can also be used to capture the perspectives of those who may have been hard to engage with more traditional research methods. These are important findings that have the potential to optimize study recruitment and retention, reduce research burden, and improve the quality and representativeness of psychosis research.

Many patients and family members agreed to receive the web-based consent form, and those who consented had high survey completion rates. Although few studies have used online recruitment and survey administration methods with this population, our completion rates for this one-time survey were substantially higher than those found in a study that delivered regular surveys through a mobile app to youth receiving EPI care [34]. Our high completion rates could be explained by the low burden and low-risk nature of the study. Thus, online methods may not be effective at engaging patients and families in research if the study is perceived as burdensome or the risk is perceived as high. IRBs should assess the risks and benefits of participating in remote research while considering decision-making capacity, stigma, and data privacy concerns [35]. It is also likely that completion rates are influenced by the study setting; in this study, the setting was an EPI program that integrates clinical care and research and uses a coordinated recruitment process that provides all patients with an opportunity to participate in research. Completion rates may also be influenced by the timing of study recruitment, in that patients and families may feel pressure to participate in research when they are entering a mental health program because of worries that their refusal may impact their care. Future studies should examine the feasibility and effectiveness of using digital research tools in more complex studies that recruit participants at different stages in their care journey or other mental health services, particularly those that may not have the infrastructure to support a robust and coordinated recruitment system.

Close to half of the patients who completed the survey were female. This is more than the average for EPI programs, in which the proportion of females is closer to one-quarter to one-third [36], and while the majority identified with racial groups other than White, the proportion (less than 60%) was lower than our patient population as a whole (closer to 70%) [37]. This suggests that further work is needed to ensure that psychosis research is representative of the population served in clinical programs.

We found that more than one-third of family members and almost one-quarter of patients completed the consent form in person. These findings suggest that providing options for in-person recruitment methods is still a meaningful strategy to engage patients with psychosis and their families in research, especially during the consent process. Family members in particular relied more on in-person support to consent to the study but almost all went on to complete the survey remotely, suggesting that once family members understand and consent to a study, there is a high likelihood that they will feel comfortable using online survey administration methods. This in-person support may also have acted as a technical orientation to REDCap. While digital tools can augment current methods of recruitment, they may also bias results to individuals from specific backgrounds. In-person engagement strategies may be particularly meaningful for urban EPI programs that serve multicultural populations with language barriers or vulnerable groups, including people experiencing homelessness, who may not have consistent access to technology [11,22,26,38].

Patients and family members in our study preferred to receive research materials by email rather than text message. This finding was somewhat surprising, given the high rates of mobile phone ownership and text messaging among young people with psychosis [16,39]. While previous studies suggested that youth with psychosis prefer text messaging and social media, our findings suggest that for the purposes of research, patients with psychosis and their families prefer to receive consent forms and surveys via email [19,20,40]. Although the reasons for this are unclear, one possibility is that online surveys are more user-friendly to complete by email using a computer or tablet than by texted link on a smartphone. Also, patients and family members may have felt that email was a more secure communication tool than text message [41]. We also found that most surveys were completed on weekday afternoons, and patients and family members preferred to receive e-gift cards from a coffee shop as study compensation. This finding highlights factors that may yield better recruitment and completion rates in future studies that engage people with psychosis and their families in research.

Similar to past studies, we had challenges recruiting patients with psychosis and their families in our digital research study and retaining participants between consenting and completing the survey. Many patients and family members did not respond to the web-based consent form and survey, some partially completed it, and one family member experienced technical difficulties. Engaging this population via phone call reminders also posed challenges, including incorrect contact information and phone numbers that were no longer in service. Similar barriers were encountered in prior studies that relied on in-person research visits and exemplify some of the difficulties in recruiting and retaining this population in research [1,11,41]. While online administration methods are feasible and acceptable among patients with psychosis and their families and may increase research engagement, challenges are still present.

These findings have timely implications in the context of the COVID-19 pandemic as the health care system navigates the shift to virtual care and remote research [42]. Digital tools such as those used in this study can facilitate the adaptation and continuation of current projects that are on hold as well as the initiation of new research. The rapid adoption of digital tools to support research amid COVID-19 has put pressure on IRBs to establish standard procedures and safety protocols for remote research, including consent and assessment frameworks. As digital tools become widely deployed to facilitate different types of research in varied populations, it is incumbent on researchers adopting them to study their uptake and safety. New frameworks for remote research and data supporting its acceptability, feasibility, and safety may allow it to persist beyond the pandemic as a way to improve research engagement.

### Strengths and Limitations of the Study

Our study has notable strengths. While digital tools are increasingly being used to support research with populations with mental illness, and particularly young people [43,44], we are not aware of studies that have specifically examined the uptake of digital research tools with people with psychosis and their families. This is an important advance given the established barriers to research recruitment in psychosis and the paucity of literature studying novel approaches to increase research engagement. This is particularly timely given the impact of the COVID-19 pandemic on clinical research across disorders, when evidence to guide the implementation of tools to conduct research remotely is so critical. Establishing feasibility of this approach to remote research in family members provides additional value, given the importance of capturing caregiver perspectives in psychosis research [45].

There are some study limitations to consider. First, while we attempted to approach all referred patients and their family members, those who were not approached or did not complete the consent form or survey may have been systematically different from survey completers. Unfortunately, we were unable to gather information on the patients and family members who did not participate. Approaching potential participants in person did allow us to reach some individuals who may otherwise have declined participation due to problems accessing a mobile device or the internet. Family member participation may have been subject to additional sampling bias if the family member was not present to verbally consent to receive the web-based consent form, as recruitment mostly relied on their in-person attendance at clinic appointments. Some additional factors may limit the generalizability of our findings, as the study was conducted at a well-resourced EPI program in Toronto, Canada, with an integrated clinical research program that may have been more conducive to innovative research methods.

### Recommendations for Conducting Remote Research in Psychosis

Our results suggest that future studies would benefit from including both in-person and virtual engagement strategies, particularly for family members who may want to discuss the study in person and require additional support from the research team. Further, prolonged engagement in the form of telephone and email reminders may be necessary to increase participation rates. Given that in-person administration may have also provided technical support, we suggest that the selected consent and survey platform be tested with a target audience before it is launched so that functionality issues can be addressed. Outlining potential usability challenges in the consent form or a tip sheet could help participants orient themselves to the platform and make the online methods more user-friendly. Consent forms and surveys may have the highest yield if they are sent to participants on weekday afternoons. Participant attrition between consent and survey completion suggests that participants completing surveys remotely may have restrictions on time, and platforms that allow them to partially complete surveys and then return to complete them later may yield higher completion rates. To continue developing and using digital research methods more broadly, researchers should build relationships with their IRBs and work together to develop ethical standards for conducting remote research safely.

### Conclusions

Digital tools offer a low-burden way of engaging people with psychosis and their families in research. These findings demonstrate that virtual consent and survey administration methods are feasible and acceptable and can be used to capture the perspectives of those who may have been hard to engage with more traditional research methods. Leveraging data on preferences and usage patterns to guide the use of a variety of digital and in-person research tools can help optimize study recruitment and retention, improving the quality and representativeness of psychosis research during the COVID-19 global pandemic and beyond.

## Appendix

Multimedia Appendix 1 Study recruitment process.

Multimedia Appendix 2 Checklist for Reporting Results of Internet E-Surveys (CHERRIES).

## Abbreviations

CHERRIES: Checklist for Reporting Results of Internet E-Surveys
e-gift: electronic gift
EPI: early psychosis intervention
IRB: institutional review board
REDCap: Research Electronic Data Capture
SCEIS: Slaight Centre Early Intervention Services
