# Supplementary figures and images for: Using Digital Tools to Engage Patients With Psychosis and Their Families in Research: Survey Recruitment and Completion in an Early Psychosis Intervention Program
Source: JMIR Ment Health. 2021 May 31;8(5):e24567. doi: 10.2196/24567 (PMC8204241; doi:10.2196/24567)

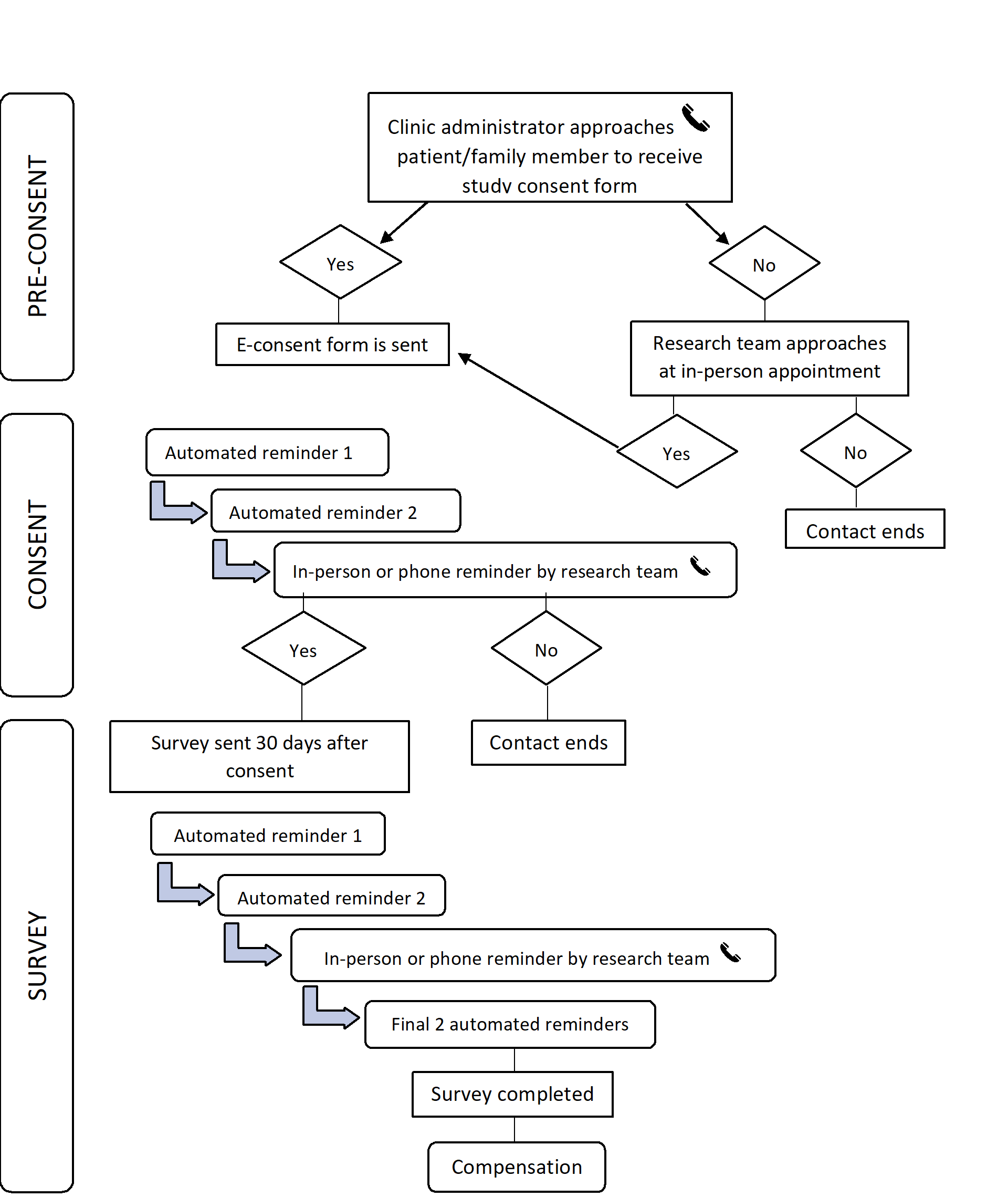

Supplement: Multimedia Appendix 1 [file mental_v8i5e24567_app1.png]
